# The Battle over mTOR: An Emerging Theatre in Host–Pathogen Immunity

**DOI:** 10.1371/journal.ppat.1002894

**Published:** 2012-09-13

**Authors:** Sunil Martin, Bhaskar Saha, James L. Riley

**Affiliations:** 1 Department of Microbiology, Abramson Family Cancer Research Institute, Perelman School of Medicine, Philadelphia, Pennsylvania, United States of America; 2 Lab #5, National Centre for Cell Science, Ganeshkhind-Pune, India; The Fox Chase Cancer Center, United States of America

Mammalian target of rapamycin (mTOR) kinases are emerging as master regulators of cellular metabolism [1]. During an infection, pathogens seek nutrition to survive and often exploit host machinery that controls cellular metabolic processes. Moreover, pathogens can subvert host metabolism by targeting mTOR complexes to gain a replicative advantage. Conversely, host cells regulate the mTOR axis to facilitate pathogen clearance. Intriguingly, in addition to their role in the regulation of metabolism, mTOR complexes regulate both the quality and quantity of innate and adaptive immune responses. Here we propose that drugs strategically targeting mTOR, perhaps in opposing ways in distinct cell types, could influence the immunological outcome of host–pathogen interactions and also act as effective antibiotics by limiting pathogen replication.

## Targeting Immune Cell Metabolism in the Complex Host Pathogen Struggle

During infections, proinflammatory and anti-inflammatory immune responses are cross regulated to achieve host protection while limiting pathologic insult [2]. This synchronized and swift mounting of an immune response is metabolically altering and demanding [1]. Innate and adaptive stimulation trigger not only activation of the immune system and proliferation of effector cells but also induce the uptake and utilization of extracellular nutrients (e.g. free fatty acids, glucose, and glutamine) and growth factors (e.g. GM-CSF, IL-2) and the release of lactic acid and other cellular waste products [3]–[5]. Immune cells sense nutritional cues from the microenvironment to switch between metabolically inactive and active stages [6]. Inflammatory mediators (TNF) and cytokines (IL-4, IL-6) can alter a cell's insulin responsiveness and may impact the general host glucose metabolism [7]. Conversely, extracellular nutrients may directly modulate immune response [8]. Pathogens often exploit host metabolic pathways and survival machineries as an adaptive strategy for their persistence and proliferation [2]. However, to date effective antibiotics targeting mTOR activity have not been developed. We opine that recent advances in both gene therapy and nanotechnology [9], [10] will enable investigators to alter mTOR activity distinctly in specific cell types, and these will become potent antibiotics by limiting pathogen replication and augmenting protective immune responses.

## mTOR Evolution…from Cell Metabolism to Host Defense?

mTOR is a serine/threonine kinase that performs fundamental roles in integrating cell growth and metabolism [1]. In the mammalian host, there are two related signaling complexes known as mTORc1 and mTORc2. The mTORc1 complex induces phosphorylation of the ribosomal protein S6 and the translation-initiation inhibitor 4E-BP1. mTORc2 regulates phosphorylation of serine 473 of Akt as well as the organization of the actin cytoskeleton [11]. In the immune cells, mTOR complexes regulate key metabolic processes of cell survival including protein synthesis, glucose and lipid metabolism, autophagy, and apoptosis [12]. Interestingly, mTOR itself is highly conserved but the signaling pathways altered by mTOR activity have evolved considerably [13]. That is, whereas amino acid sequences of the core mTOR signaling proteins are well conserved, the signaling nodes that make use of the mTOR complexes have evolved considerably as eukaryotes have transitioned from single-cell organisms to humans. For instance, signaling pathways initiated by insulin and TNFα binding, which evolutionarily developed after mTOR, have co-opted parts of mTOR signaling machinery to deliver their biological effect. Thus, the mTOR core assumed more responsibilities without adding more functional domains. In addition to evolutionary pressures, it is tempting to speculate that pathogens also played a role in the evolution of mTOR signaling. Since initially the main role of mTOR was to regulate cellular metabolism, it was well positioned to sense invading microbes that hijacked cellular metabolism to feed their own replication. Thus as immune systems developed, the signaling pathways that regulated immunity also took advantage of the mTOR pathway as a means to detect and control pathogen replication, and as such pathogens have targeted this pathway as a means to ensure their survival and replication. Table 1 provides a list of pathogens and their targets in the mTOR signaling cascade. For instance, initial interactions between invading pathogens and dendritic cells (DCs) initiate a sequence of events leading to DC maturation and migration to the draining lymph nodes [14]. However, many invading pathogens have devised strategies to induce DC paralysis by targeting key signal transduction pathways such as PI3 kinase-Akt-mTOR pathways. HIV-1 activates mTOR through HIV-1_ENV_-mediated signal transduction, which impedes autophagy in mucosal DCs, resulting in reduced autophagosome degradation, less processing and presentation of HIV-1 antigens, and enhanced viral transfer from DC to CD4^+^ T cells [15]. Likewise, herpes simplex virus 1 uses viral kinase Us3, a functional surrogate of Akt, to phosphorylate tuberous sclerosis complex 2 (TSC2), constitutively activating mTORc1 and circumventing S6K-mediated feedback inhibition to enhance production of viral proteins [16]. In EBV triggered lymphomas, rapamycin is able to serve a dual role as protector against GVHD and as an antitumor agent by limiting an IL-10 autocrine growth pathway, resulting in less tumor growth [17]. Along the same lines, bacterial infections *(Staphylococcus aureus*, *Listeria monocytogenes)* are known to activate the mTOR complexes in DCs, triggering the production of the anti-inflammatory cytokine IL-10 and thereby promoting their survival in the host [18]. Conversely, inhibition of mTOR in APCs was found to induce IL-12 production and robust T helper 1 (Th1) and Th17 polarization, thus facilitating pathogen clearance [19]. Blocking mTORc1 in macrophages increases IL-12 production and decreases IL-10 production during infections with *Leishmania donovani* and *Leishmania major*. Toxoplasma gondii targeting of mTORc2 may limit the mobility of the host cell and may prevent the spreading of the infection [20]. Hence, based on the timing, cell type, and pathogen, alterations to mTOR signaling can have beneficial or harmful consequences for the host.

**Table 1:** DNA viruses that target mTOR signaling pathways.

Molecular Targets	Viruses or Viral Proteins	References
PI3 kinase*	PyV (Py-middle tumor antigen), HPV (HP-virus-like particles)	[30], [31]
PP2A (inhibits Akt activation)	polyomavirus small T-antigen, simian virus small tumor antigen, human papillomavirus E7	[32]–[34]
Akt (PKB)*	myxoma virus ankyrin repeat, host range factor M-T5, herpes simplex virus-1 uses viral kinase Us3	[16], [35], [36]
mTORc2*	HCMV	[37]
AMPK (inhibits TSC)*	HCMV	[38]
TSC (inhibits Rheb-GTP)	human papillomavirus E6 oncoprotein (HPV-E6)	[34], [39]
mTORc1*	HIV-1 *Env,* HCMV	[15], [40]
PP2A (which activates 4E-BP)	simian virus small tumor antigen (SVST), adenovirus E4-ORF4	[41], [42]
4E-BP (inhibits the eLF4F complex)	HSV, VV	[43], [44]
eIF4F complex*	HCMV, simplex virus protein ICP0, VV	[43], [45], [46]

* Virus activates the target. Unmarked, virus inhibits the target. **PKB**, Protein Kinase B; **AMPK**, AMP-activated kinase; **TSC**, tuberous sclerosis complex; **PP2A**, Protein Phosphatase 2A; **4E-BP**, eIF4E binding protein; **elF4F**, eukaryotic elongation factor complex consisting of elF4E, elF4G, elF4A, and MnK1; **PyV**, polyomavirus; **HPV**, human papillomavirus; **HCMV**, human cytomegalovirus; **HSV**, herpes simplex virus; **VV**, vaccinia virus.

**Figure 1:**
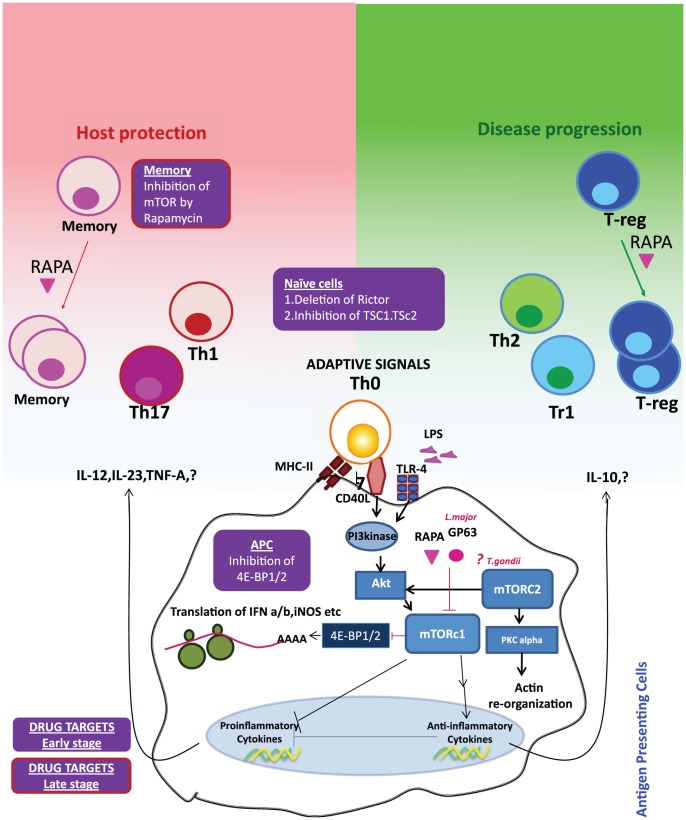
Reciprocal consequences of mTOR activation in APCs and T cells may be host protective or disease promotive. Innate (e.g. TLRs) or adaptive signals (e.g. CD40) trigger the PI3 kinase-Akt-mTOR signaling cascade in the APCs. Activation of mTORc1 leads to the phosphorylation of 4E-BP1/2 and initiation of protein translation. Pathogenic virulence factors such as Gp63 and antibiotic rapamycin (RAPA) inhibit mTOR activation and hence downregulate translation of type I interferons and iNOS (inducible nitric oxide synthase). Inhibition of 4E-BP1/2 can selectively upregulate translation and hence may be an attractive drug target. mTOR activation can also upregulate anti-inflammatory molecule IL-10 and inhibits the proinflammatory molecules, such as IL-12. IL-10 may skew Th0 cells to the disease-promoting Th2/Treg cells, whereas IL-12 and other proinflammatory cytokines can enhance the Th1/Th17 axis. Activation of mTOR signaling by inhibition of TSC1/TSC2 (tuberous sclerosis complex) or inhibition of Rictor (rapamycin-insensitive companion of mTOR, an essential component of mTORc1 signaling), especially at the early stage of an infection, can boost the propensity of these cells to be skewed towards Th1 phenotype. mTOR inhibition of Treg cells by rapamycin can augment expansion of Treg cells with increased suppressive capacity. This can be prevented by the activation of mTOR by inhibiting TSC1/2 or PTEN (Phosphatase and TENsin homolog) and may be a lucrative drug target at the later stages of an infection. On the other hand, inhibition of mTOR signaling in memory cells can improve the memory cell differentiation. Blockade of mTOR by pharmacological and genetic ablation enhances the quality and quantity of surviving memory. Targeted inhibition of mTOR in memory cells can thus be an attractive drug target especially at the later stage of infection.

Studies of *Leishmania* infection in macrophages have shown that virulence factors directly modulate mTOR stability [21]. The leishmanial metallo-glycoproteinase Gp63 cleaves the mTOR complex to downregulate the host translational machinery by activating the transcriptional repressor 4-EBP1. This results in substantially reduced production of the host protective type I interferon (IFNα/IFN-β) and iNOS (induced nitric oxide synthase). Nevertheless, mTOR inhibition could possibly enhance the host protective IL-12 production and impede pathogen-supportive IL-10 production, which would give rise to Th1 and Th2/Treg responses, respectively [22]. Thus, pathogens seek survival within the host cells while host cells link mTOR signaling to a pathogen-clearance mechanism (Figure 1). Together, these observations indicate that pathogens have evolved to target mTOR, the key metabolic spigot of the APCs. However, mTOR inhibition is wired to a host protective “proinflammatory program” in the host APCs, which reverses signals to amplify antimicrobial adaptive immune response.

## Paradigms and Perils of the Therapeutic Targeting of mTOR

We speculate that many potential pathogen targets exist which could be influenced for the benefit of the host by modulating mTOR signaling. Robust generation of the type I interferons (IFN-α and IFN-β) from DCs is one of the crucial early innate antiviral immune responses. It has been demonstrated that blocking mTOR or its downstream signaling molecules impairs the production of type I interferon from plasmacytoid DCs; thus, preemptive activation of mTOR signaling in DCs could stop many infections before they get a chance to establish a foothold. However, blocking mTOR signaling could be beneficial for the replication of therapeutic oncolytic viruses, which are sensitive to type I interferon [23]. Additionally since mTORc1 activation enhances lipid biosynthesis [24] (which is associated with host resistance to *Leishmania* infection [25]), it is reasonable to speculate that activation of PPAR-γ and SREBP (which link mTOR to fatty acid metabolism) could be an effective strategy for increasing fatty acid production during infection, as this increase in lipid biosynthesis would in turn impair parasite survival. Thus, selective and rational targeting of mTOR or its downstream signaling pathways linking to specific metabolic pathways in DCs can enhance the overall efficacy of this approach.

mTOR complexes are also implicated in the generation of durable and functional antimicrobial memory T cell responses. Rapamycin administration leads to an enhancement of the quality and quantity of memory T cell responses in the lymphocytic choriomeningitis virus (LCMV) model [26]. A recent study that examined the distinct downstream transcriptional programs of mTOR in a tumor model has pinpointed mTOR as a determinant in effector versus memory CD8^+^ T cell fate [27]. Clarifying whether memory T cell generation is synergistically or reciprocally regulated through mTORc1 and mTORc2 will help to further fine-tune the ideal target for therapeutic intervention. To better understand the immunological and metabolic conundrum of mTOR, an integrated view of the mTOR-regulated lymphokine expression and surface molecular expression on APCs and T cells during an infection would produce insight into the right target and timing of the therapeutic intervention. It remains to be seen mechanistically, immunologically, and biochemically whether targeting the mTORc1 and mTORc2 complexes with pathogen-derived factors such as glycoproteins will shift the balance between proinflammatory and anti-inflammatory T cell responses in favor of pathogen survival.

In the context of the apparently contrasting immunological outcomes of mTOR's function in T cells and APCs [28], designing drugs capable of modulating the mTOR signaling axis to fight against infectious diseases becomes especially challenging. For instance, in leishmaniasis the primary effect of mTOR blockade in APCs by the metalloprotease Gp63 is to reduce translation by activating the translational repressor 4E-BP1. However, mTOR inhibition within T cells could force them to differentiate into Treg/Th2 cells, which would likely perpetuate the infection. Therefore, we envision a targeting regime that involves the simultaneous inhibition of 4E-BP1 in APCs and the activation of mTOR in CD4 T cells to enhance the host protective Th1 response in the early stages of infection. Since the hallmark of a durable anti-leishmanial immune response is an augmentation of the quality and quantity of memory T cells, we propose that the pathogen-specific recall responses can be enhanced by targeting mTOR inhibitors to the memory cells in vivo to enhance their differentiation. Treg-specific activation of mTOR by inhibiting upstream inhibitors of mTOR signaling such as TSC1/TSC2 or PTEN can reduce the Foxp3 expression and may break tolerance. Targeting memory cells and Treg cells can be especially beneficial during the later stages of infection. By extension, use of metalloprotease Gp63, either alone or in combination with other drugs, may offer scope for rational therapeutic interventions against autoimmune diseases such as multiple sclerosis, wherein type I interferons aggravate the disease [29].

## Conclusions and Perspectives

Pathogens are armed with virulence factors that target mTOR to manipulate the host towards a unique metabolic state with immunological outcomes that favor pathogen survival. In contrast, there are few effective antibiotics that alter the host metabolism to favor pathogen clearance. One reason for this is that systematic delivery of metabolism-altering agents such as rapamycin often has opposing actions: they limit pathogen replication but also alter the immune response to limit pathogen clearance. We propose the design of drugs that promote distinct and likely contradictory immunological changes in the APC and T cells. For instance, the optimal therapy to combat *Leishmania* would activate mTOR in macrophages and effector T cells while suppressing mTOR in T cells destined to become memory cells. This would limit pathogen replication, promote immunity, and generate protective memory. This therapeutic modality of reversion of propathogenic metabolic phenotype may lead to host protection that could rightly be called “immuno-metabolic therapy.” Thus, the differential host and pathogen, immune cell type-specific and disease phase–specific functions of mTOR represent a conundrum that must be carefully considered.

## References

[1] Delgoffe GM, Powell JD (2009) mTOR: taking cues from the immune microenvironment. Immunology 127: 459–465. 19604300 10.1111/j.1365-2567.2009.03125.x PMC2729523

[2] Bhavsar AP, Guttman JA, Finlay BB (2007) Manipulation of host-cell pathways by bacterial pathogens. Nature 449: 827–834. 17943119 10.1038/nature06247

[3] Frauwirth KA, Riley JL, Harris MH, Parry RV, Rathmell JC, et al (2002) The CD28 signaling pathway regulates glucose metabolism. Immunity 16: 769–777. 12121659 10.1016/s1074-7613(02)00323-0

[4] Vander Heiden MG, Plas DR, Rathmell JC, Fox CJ, Harris MH, et al (2001) Growth factors can influence cell growth and survival through effects on glucose metabolism. Mol Cell Biol 21: 5899–5912. 11486029 10.1128/MCB.21.17.5899-5912.2001 PMC87309

[5] Wada HG, Indelicato SR, Meyer L, Kitamura T, Miyajima A, et al (1993) GM-CSF triggers a rapid, glucose dependent extracellular acidification by TF-1 cells: evidence for sodium/proton antiporter and PKC mediated activation of acid production. J Cell Physiol 154: 129–138. 7678263 10.1002/jcp.1041540116

[6] Fox CJ, Hammerman PS, Thompson CB (2005) Fuel feeds function: energy metabolism and the T-cell response. Nat Rev Immunol 5: 844–852. 16239903 10.1038/nri1710

[7] Winer S, Winer DA (2012) The adaptive immune system as a fundamental regulator of adipose tissue inflammation and insulin resistance. Immunol Cell Biol 10.1038/icb.2011.110 22231651

[8] Kau AL, Ahern PP, Griffin NW, Goodman AL, Gordon JI (2011) Human nutrition, the gut microbiome and the immune system. Nature 474: 327–336. 21677749 10.1038/nature10213 PMC3298082

[9] June CH, Blazar BR, Riley JL (2009) Engineering lymphocyte subsets: tools, trials and tribulations. Nat Rev Immunol 9: 704–716. 19859065 10.1038/nri2635 PMC3412112

[10] Zhang Y, Satterlee A, Huang L (2012) In Vivo Gene Delivery by Nonviral Vectors: Overcoming Hurdles[quest]. Mol Ther 20: 1298–1304. 22525514 10.1038/mt.2012.79 PMC3392980

[11] Zoncu R, Efeyan A, Sabatini DM (2011) mTOR: from growth signal integration to cancer, diabetes and ageing. Nat Rev Mol Cell Biol 12: 21–35. 21157483 10.1038/nrm3025 PMC3390257

[12] Powell JD, Pollizzi KN, Heikamp EB, Horton MR (2012) Regulation of Immune Responses by mTOR. Annu Rev Immunol 30: 39–68. 22136167 10.1146/annurev-immunol-020711-075024 PMC3616892

[13] van Dam TJ, Zwartkruis FJ, Bos JL, Snel B (2011) Evolution of the TOR pathway. J Mol Evol 73: 209–220. 22057117 10.1007/s00239-011-9469-9 PMC3236823

[14] Gougeon ML, Melki MT, Saidi H (2012) HMGB1, an alarmin promoting HIV dissemination and latency in dendritic cells. Cell Death Differ 19: 96–106. 22033335 10.1038/cdd.2011.134 PMC3252828

[15] Blanchet FP, Moris A, Nikolic DS, Lehmann M, Cardinaud S, et al (2010) Human immunodeficiency virus-1 inhibition of immunoamphisomes in dendritic cells impairs early innate and adaptive immune responses. Immunity 32: 654–669. 20451412 10.1016/j.immuni.2010.04.011 PMC2929482

[16] Chuluunbaatar U, Roller R, Feldman ME, Brown S, Shokat KM, et al (2010) Constitutive mTORC1 activation by a herpesvirus Akt surrogate stimulates mRNA translation and viral replication. Genes Dev 24: 2627–2639. 21123650 10.1101/gad.1978310 PMC2994037

[17] Nepomuceno RR, Balatoni CE, Natkunam Y, Snow AL, Krams SM, et al (2003) Rapamycin inhibits the interleukin 10 signal transduction pathway and the growth of Epstein Barr virus B-cell lymphomas. Cancer Res 63: 4472–4480. 12907620

[18] Weichhart T, Costantino G, Poglitsch M, Rosner M, Zeyda M, et al (2008) The TSC-mTOR signaling pathway regulates the innate inflammatory response. Immunity 29: 565–577. 18848473 10.1016/j.immuni.2008.08.012

[19] Delgoffe GM, Pollizzi KN, Waickman AT, Heikamp E, Meyers DJ, et al (2011) The kinase mTOR regulates the differentiation of helper T cells through the selective activation of signaling by mTORC1 and mTORC2. Nat Immunol 12: 295–303. 21358638 10.1038/ni.2005 PMC3077821

[20] Wang Y, Weiss LM, Orlofsky A (2010) Coordinate control of host centrosome position, organelle distribution, and migratory response by Toxoplasma gondii via host mTORC2. J Biol Chem 285: 15611–15618. 20236941 10.1074/jbc.M109.095778 PMC2865287

[21] Jaramillo M, Gomez MA, Larsson O, Shio MT, Topisirovic I, et al (2011) Leishmania repression of host translation through mTOR cleavage is required for parasite survival and infection. Cell Host Microbe 9: 331–341. 21501832 10.1016/j.chom.2011.03.008

[22] Mathur RK, Awasthi A, Wadhone P, Ramanamurthy B, Saha B (2004) Reciprocal CD40 signals through p38MAPK and ERK-1/2 induce counteracting immune responses. Nat Med 10: 540–544. 15107845 10.1038/nm1045

[23] Alain T, Lun X, Martineau Y, Sean P, Pulendran B, et al (2010) Vesicular stomatitis virus oncolysis is potentiated by impairing mTORC1-dependent type I IFN production. Proc Natl Acad Sci U S A 107: 1576–1581. 20080710 10.1073/pnas.0912344107 PMC2824402

[24] Peterson TR, Sengupta SS, Harris TE, Carmack AE, Kang SA, et al (2011) mTOR complex 1 regulates lipin 1 localization to control the SREBP pathway. Cell 146: 408–420. 21816276 10.1016/j.cell.2011.06.034 PMC3336367

[25] Rub A, Dey R, Jadhav M, Kamat R, Chakkaramakkil S, et al (2009) Cholesterol depletion associated with Leishmania major infection alters macrophage CD40 signalosome composition and effector function. Nat Immunol 10: 273–280. 19198591 10.1038/ni.1705

[26] Araki K, Turner AP, Shaffer VO, Gangappa S, Keller SA, et al (2009) mTOR regulates memory CD8 T-cell differentiation. Nature 460: 108–112. 19543266 10.1038/nature08155 PMC2710807

[27] Li Q, Rao RR, Araki K, Pollizzi K, Odunsi K, et al (2011) A central role for mTOR kinase in homeostatic proliferation induced CD8+ T cell memory and tumor immunity. Immunity 34: 541–553. 21511183 10.1016/j.immuni.2011.04.006 PMC3083826

[28] Weichhart T, Saemann MD (2009) The multiple facets of mTOR in immunity. Trends Immunol 30: 218–226. 19362054 10.1016/j.it.2009.02.002

[29] Axtell RC, de Jong BA, Boniface K, van der Voort LF, Bhat R, et al (2010) T helper type 1 and 17 cells determine efficacy of interferon-beta in multiple sclerosis and experimental encephalomyelitis. Nat Med 16: 406–412. 20348925 10.1038/nm.2110 PMC3042885

[30] Meili R, Cron P, Hemmings BA, Ballmer-Hofer K (1998) Protein kinase B/Akt is activated by polyomavirus middle-T antigen via a phosphatidylinositol 3-kinase-dependent mechanism. Oncogene 16: 903–907. 9484781 10.1038/sj.onc.1201605

[31] Fothergill T, McMillan NA (2006) Papillomavirus virus-like particles activate the PI3-kinase pathway via alpha-6 beta-4 integrin upon binding. Virology 352: 319–328. 16781758 10.1016/j.virol.2006.05.002

[32] Andrabi S, Gjoerup OV, Kean JA, Roberts TM, Schaffhausen B (2007) Protein phosphatase 2A regulates life and death decisions via Akt in a context-dependent manner. Proc Natl Acad Sci U S A 104: 19011–19016. 18006659 10.1073/pnas.0706696104 PMC2141899

[33] Pipas JM, Levine AJ (2001) Role of T antigen interactions with p53 in tumorigenesis. Semin Cancer Biol 11: 23–30. 11243896 10.1006/scbi.2000.0343

[34] Pim D, Massimi P, Dilworth SM, Banks L (2005) Activation of the protein kinase B pathway by the HPV-16 E7 oncoprotein occurs through a mechanism involving interaction with PP2A. Oncogene 24: 7830–7838. 16044149 10.1038/sj.onc.1208935

[35] Wang G, Barrett JW, Stanford M, Werden SJ, Johnston JB, et al (2006) Infection of human cancer cells with myxoma virus requires Akt activation via interaction with a viral ankyrin-repeat host range factor. Proc Natl Acad Sci U S A 103: 4640–4645. 16537421 10.1073/pnas.0509341103 PMC1450224

[36] Stanford MM, Barrett JW, Nazarian SH, Werden S, McFadden G (2007) Oncolytic virotherapy synergism with signaling inhibitors: Rapamycin increases myxoma virus tropism for human tumor cells. J Virol 81: 1251–1260. 17108021 10.1128/JVI.01408-06 PMC1797522

[37] Clippinger AJ, Maguire TG, Alwine JC (2011) The changing role of mTOR kinase in the maintenance of protein synthesis during human cytomegalovirus infection. J Virol 85: 3930–3939. 21307192 10.1128/JVI.01913-10 PMC3126115

[38] Kudchodkar SB, Del Prete GQ, Maguire TG, Alwine JC (2007) AMPK-mediated inhibition of mTOR kinase is circumvented during immediate-early times of human cytomegalovirus infection. J Virol 81: 3649–3651. 17215282 10.1128/JVI.02079-06 PMC1866081

[39] Lu Z, Hu X, Li Y, Zheng L, Zhou Y, et al (2004) Human papillomavirus 16 E6 oncoprotein interferences with insulin signaling pathway by binding to tuberin. J Biol Chem 279: 35664–35670. 15175323 10.1074/jbc.M403385200

[40] Clippinger AJ, Maguire TG, Alwine JC (2011) Human Cytomegalovirus Infection Maintains mTOR Activity and Its Perinuclear Localization during Amino Acid Deprivation. J Virol 85: 9369–9376. 21734039 10.1128/JVI.05102-11 PMC3165763

[41] Yu Y, Kudchodkar SB, Alwine JC (2005) Effects of simian virus 40 large and small tumor antigens on mammalian target of rapamycin signaling: small tumor antigen mediates hypophosphorylation of eIF4E-binding protein 1 late in infection. J Virol 79: 6882–6889. 15890927 10.1128/JVI.79.11.6882-6889.2005 PMC1112164

[42] O'Shea C, Klupsch K, Choi S, Bagus B, Soria C, et al (2005) Adenoviral proteins mimic nutrient/growth signals to activate the mTOR pathway for viral replication. EMBO J 24: 1211–1221. 15775987 10.1038/sj.emboj.7600597 PMC556401

[43] Walsh D, Mohr I (2004) Phosphorylation of eIF4E by Mnk-1 enhances HSV-1 translation and replication in quiescent cells. Genes Dev 18: 660–672. 15075293 10.1101/gad.1185304 PMC387241

[44] Walsh D, Arias C, Perez C, Halladin D, Escandon M, et al (2008) Eukaryotic translation initiation factor 4F architectural alterations accompany translation initiation factor redistribution in poxvirus-infected cells. Mol Cell Biol 28: 2648–2658. 18250159 10.1128/MCB.01631-07 PMC2293122

[45] Walsh D, Perez C, Notary J, Mohr I (2005) Regulation of the translation initiation factor eIF4F by multiple mechanisms in human cytomegalovirus-infected cells. J Virol 79: 8057–8064. 15956551 10.1128/JVI.79.13.8057-8064.2005 PMC1143722

[46] McMahon R, Zaborowska I, Walsh D (2011) Noncytotoxic inhibition of viral infection through eIF4F-independent suppression of translation by 4EGi-1. J Virol 85: 853–864. 21068241 10.1128/JVI.01873-10 PMC3019991

